# Injectable Contraceptives as an Underutilized Option for Women's Reproductive Health: An Exploratory Qualitative Study

**DOI:** 10.7759/cureus.65576

**Published:** 2024-07-28

**Authors:** Subalakshmi Subramaniyan, Madhivanan Arulmozhi, Kalaiselvan Ganapathy, Reenaa Mohan

**Affiliations:** 1 Community Medicine, Sri Manakula Vinayagar Medical college and Hospital, Puducherry, IND; 2 Community Medicine, Sri Manakula Vinayagar Medical College and Hospital, Puducherry, IND; 3 Community and Family Medicine, All India Institute of Medical Sciences Mangalagiri, Guntur, IND

**Keywords:** solutions, barriers, perspectives, qualitative, dmpa, injectable contraception

## Abstract

Background: Injectable contraceptives (IC) provide a highly effective, reversible method of preventing conception, yet discontinuation rates are high. Health workers play a crucial role in the successful implementation of family welfare services. Adding up the basket of choices without knowing the community's needs can lead to poor utilization of services.

Objectives: To explore the facilitators and barriers to the utility of injectable contraceptives among reproductive women from the user’s point of view and to understand solutions from the key informants.

Methodology: The study was conducted in the field practice areas among reproductive women attending a primary health center in Puducherry. It was an exploratory qualitative study in which in-depth interviews were conducted among 19 IC users using an interview guide. This was followed by a key informant interview with seven service providers, including doctors, staff nurses, auxiliary nurse midwives, and Anganwadi workers, to explore the solutions for the identified barriers. Purposive, convenient sampling was adopted for the selection of study participants, and the sample size was chosen until the point of saturation. Two investigators trained in qualitative research have performed a manual content analysis of transcripts to ensure credibility. Descriptive codes were derived, and similar codes were merged into categories and themes.

Results: The most common facilitators were awareness from service providers and dissatisfaction with previous methods. Fear of side effects, inadequate information, out-of-pocket expenditure, lack of family support, and sociocultural myths were the most common barriers. Key informants suggested counseling on side effects, incentive-based follow-up, universal health insurance, couple-based counseling, training of service providers, and a positive deviance approach.

Conclusion: Health workers are pivotal in the successful delivery of family welfare services. The acceptability of IC could be improved by addressing concerns about side effects and its effective management through various targeted interventions.

## Introduction

Worldwide, 214 million reproductive-age women have an unmet need for contraception due to multifaceted reasons such as less accessibility, a limited range of available contraceptive methods, fear of side effects, opposition to cultural beliefs, and gender-based barriers [1]. Generally, a single predominant method limits the use of modern contraceptive methods and increases unmet needs for contraception. The practice of a wide range of contraceptive choices promotes the acceptance and continuation rate, increasing the overall prevalence of contraceptive use [2]. Effective family planning with birth spacing methods could avert 30% of maternal deaths and 10% of child deaths, including unintended pregnancies and unsafe abortions [3]. In India, the contraceptive prevalence rate among eligible couples was 75%, with 56.5% of women using modern contraceptives, as per the National Family Health Survey (NFHS-5). Of these, tubectomy accounts for 37.9%, condoms for 9.5%, oral pills for 5.1%, intrauterine devices for 2.1%, and only 0.6% use injectable contraceptives. Notably, in Puducherry, only 0.4% use injectable contraceptives [4].

In 2016, injectable contraceptives were included in India’s National Family Planning Program as ‘Antara', a three-monthly injection, under the brand of Depot Medroxy Progesterone Acetate (DMPA) by the Ministry of Health and Family Welfare. It is one of the most effective, safe, long-acting, reversible, and acceptable modern contraceptive methods, with reduced visits [3]. Apart from the birth control action, DMPA reduces the risk of endometrial and ovarian cancer, iron-deficiency anemia, pelvic inflammatory disease, ectopic pregnancy, and fibroids. It can be used safely in breast-feeding women, people with cerebrovascular disease, migraine headaches, congestive heart failure, coronary artery disease, lipid disorders, peripheral vascular disease, and systemic lupus erythematosus [5,6].

For any health program with newer initiatives, it is crucial to assess the effective utilization and address the gaps for its successful implementation. Adding up the basket of choices without knowing the community's needs can lead to poor utilization of services. It has been found that discontinuation rates were high among IC users after receiving the initial injection, especially for the second and third doses of DMPA [4,7]. Menstrual changes were the most frequent cause of dissatisfaction and discontinuation of DMPA [8-10]. Further, women in rural villages with improper knowledge and guidance about injectable contraceptives showed low utility of the services.

Although there have been some studies in India examining the knowledge, attitude, and practices of injectable contraceptives, the challenges and barriers faced by healthcare workers and clients in South India remain unaddressed. As the choice of contraception is a sensitive issue and has been largely influenced by sociocultural factors, we undertook a qualitative study to gain insights from participants' experiences with the following objectives: To explore the facilitators and barriers to the utility of injectable contraceptives among women of reproductive age groups from both the user's and service provider’s points of view.

## Materials and methods

This exploratory qualitative study, using in-depth interviews (IDI) and key informant interview (KII) techniques, was carried out in the field practice areas of Thirubhuvanai Primary Health Centre (PHC), attached to the Department of Community Medicine at Sri Manakula Vinayagar Medical College, a tertiary care hospital in Puducherry. This model rural PHC has two beds and serves as an upgraded health and wellness center. It provides comprehensive primary care services to around 500 outpatients daily. The PHC is well-equipped with facilities such as labs, a labor room, a pharmacy, a minor operation theater, an injection room, and a dressing room. It covers a total population of 37,041 and encompasses 14 villages and four subcentres, with 13,491 females and 6254 eligible couples. People residing in this area are provided with free family planning services from the PHC and sub-centers.

The women of reproductive age (18-49 years) residing in the study area who have ever used injectable contraception (a current user or have discontinued) services from the PHC or subcenter were included in the study. Women who were suffering from chronic diseases and those unwilling to participate in the study were excluded. The necessary Institutional Ethics Committee approval (EC number: EC/14/2022) was obtained before the commencement of the study.

We conducted in-depth interviews with 19 beneficiaries of IC users to understand the factors influencing the use and non-use of injectable contraceptives and the perceived barriers to their uptake. For the key informant interviews, we included seven service providers who have expertise in field services to obtain possible solutions for the barriers. These service providers comprised medical officers, obstetricians and gynecologists, auxiliary nurse midwives (ANMs), accredited social health activists (ASHAs), and Anganwadi workers.

We employed purposive convenience sampling to select the study participants. Purposively, we included women who provided consent and utilized injectable contraception services at the PHC. Additionally, key informants from the same PHC were recruited as they regularly interacted with the study population, aiming to gather rich information. Convenient sampling was implemented based on their availability and accessibility. The IC register at the PHC enlisted 19 women of reproductive age (18-49 years) using injectable contraception. As there is no fixed sample size for a qualitative study, based on feasibility, we interviewed all 19 eligible participants, even though saturation occurred after the 12th interview. No new information was gathered from additional interviews, indicating that data collection has reached a point of information redundancy. Successive interviews revealed a repetitive and consistent pattern of information.

Data collection procedure

After obtaining consent from the PHC medical officer, the house addresses and phone numbers of the beneficiaries were retrieved from the injectable contraception register. Subsequently, we contacted every individual by phone to determine their availability and feasibility for the interview, and the venue was planned accordingly. The female principal investigator trained in qualitative research, accompanied by an ANM, paid house visits to establish rapport and gain confidentiality with the respondents. As the investigator from the community medicine department had prior experience working in the PHC during rotation postings, it was much easier to acquaint themselves with the study participants. Participants were informed of the purpose of the study, and written consent was obtained, outlining their autonomy and privacy protections. A face-to-face interview was conducted using an interview guide that was well prepared in advance, with open-ended questions such as “What are the facilitating factors that made you choose injectable contraception?” “What are the challenges and barriers to using injectable contraceptives?” and "What, according to you, will be the possible solutions to overcome the perceived barriers?” (Appendix-1). All interviews took place in the participants' homes after ensuring privacy. Confidentiality was emphasized, assuring participants that their responses would remain anonymous. Any concerns or questions related to injectable contraception raised by the participants were addressed.

After completing the in-depth interviews with the beneficiaries, key informant interviews were conducted with the service providers. These KIIs involved broad, open-ended questions to explore possible solutions from the facilitator's point of view. The key informants were informed about the topic in advance, and interviews were scheduled at convenient times and locations. After obtaining permission, the entire conversation was audio recorded during the interview, and field notes were taken simultaneously. Each interview lasted approximately 35 to 50 minutes. Post-interview, debriefing was done with all participants. Additionally, member checking was carried out among stakeholders to validate the accuracy of the information gathered. The ethical principles, including privacy, were adhered to throughout the study.

Data analysis

The audio recordings were transcribed verbatim into English on the same day of the interview. These transcripts were meticulously checked for errors, and personal identifiers such as name and address were removed to maintain anonymity. Manual content analysis of the transcripts was done, involving coding and theme development [11]. The transcripts were read line by line repeatedly to extract the participant's meanings, and the descriptive codes were inducted. Similar codes were grouped into categories, eliminating overlapping codes. Similar categories were merged to form broad themes that depicted multiple perspectives. To enhance study validity, we triangulated multiple data sources by corroborating information from participants and key informants. The first two authors independently coded the transcripts, and the codes were cross-verified for inter-rater reliability. The final results were reviewed by the fourth author to confirm the codes and enhance the credibility of the findings. Any discrepancy arising between the investigators was resolved by mutual consensus. The study findings have been reported using “consolidated criteria for reporting qualitative research” (COREQ) guidelines [12].

## Results

The mean age of the 19 contraceptive users was 26 ± 4.57 years. Table 1 shows the socio-demographic details of the beneficiaries in the study. Of the total 19 women, the majority (15, 78.4%) were below 30 years of age, and the remaining four women (21.1%) belonged to the age group older than 30 years. About 18 participants (94.74%) were Hindu by religion, and only one (5.26%) belonged to the Christian religion. All the respondents were literate; about 16 participants (84.21%) finished their higher secondary school education, and three (15.79%) completed their graduation. The majority of 18 women (94.74%) were homemakers, and one woman was a staff nurse (5.26%) by occupation. As per the modified BG Prasad classification 2020, about nine users (47.37%) belonged to lower-middle-class socio-economic status, seven (36.84%) fell under the upper-lower class, and the remaining three (15.79%) were categorized into the upper-middle-class category. About 12 (63.16%) users were living with more than one child, and seven (36.84%) had one child. 

**Table 1:** Socio-demographic characteristics of injectable contraceptive beneficiaries (n=19)

Variables	Frequency n (%)
Age (in years)	
<30	15 (78.94)
≥30	4 (21.06)
Religion	
Hindu	18 (94.74)
Christian	1 (5.26)
Education	
Higher Secondary	16 (84.21)
Graduate	3 (15.79)
Occupation	
Homemaker	18 (94.74)
Working	1 (5.26)
Socio-economic status (as per Modified BG Prasad classification 2020)	
Upper-middle	3 (15.79)
Lower-middle	9 (47.37)
Upper-lower	7 (36.84)
Number of living children	
One child	7 (36.84)
More than one child	12 (63.16)

Table 2 shows the details of injectable contraceptive usage by the study participants. Out of a total of 19 IC ever users, the majority (16, 84.21%) discontinued injectable contraception, of which four adopted permanent methods of sterilization. Only three women (15.79%) were current users of DMPA. Of the 16 past users, six (37.5%) of women discontinued after the initial dose, three (18.75%) continued only up to the second dose, and seven (43.75%) discontinued after the third dose of DMPA. 

**Table 2:** Details of injectable contraception usage among the beneficiaries (n=19) DMPA: Depot Medroxy Progesterone Acetate

Variables	Frequency n (%)
DMPA usage	
Current user	3 (15.79)
Past user	16 (84.21)
Discontinuation rate	
After 1^st^ dose	6 (37.50)
After 2^nd^ dose	3 (18.75)
≥3^rd^ dose	7 (43.75)

Perspective of injectable contraceptive users (n=19)

The promoting factors for the use of injectable contraception among the beneficiaries were: 1) Suggestions made by the skilled service providers (staff nurse, ANM/ASHA) - [nearly 90% of the users accepted DMPA]: Nearly 15 users stated, “I came to know about this injection from my area sister during my post-natal visit and at the time of vaccination for my child in PHC”. The remaining users heard from their friends and neighbors who were current/past users of IC. 2) Discomfort in the previous method of contraception (usage of Cu-T, OCPs) (5%). 3) Safety, long duration of action (once every three months), privacy, and confidentiality (5%).

The study explored the perspectives of IC users, their barriers (Table 3), and solutions (Table 4) for IC usage from service providers. We found that lack of knowledge about IC, side effects associated with IC, sociocultural myths and misconceptions, and lack of support from family were the challenges and barriers to IC usage.

**Table 3:** Barriers to injectable contraception usage by the participants (n=19) IC: Injectable contraceptives

Themes	Categories	Codes
Individual factors	Fear of side effects	Inter-menstrual bleeding (11)
		Lethargy (10)
		Menorrhagia (7)
		Amenorrhea (5)
		Mood swings (5)
		Weight gain (3)
	Lack of Awareness	Lack of information about the choice of injectable contraception
		Concerns about adverse effects and societal judgment.
		Lack of counseling about the side-effects
		Concern about the effects of IC on fertility in future conception
	Fertility preferences	Planning for pregnancy
		Adopted a permanent method of sterilization
Family factors	Economic problems	Increased hospital visits for treatment of side-effects
		Out-of-pocket expenditure for the treatment costs.
	Family insecurity	Lack of support from the partner and in-laws.
		Difficulty in managing household activities and child-rearing due to side effects
Community factors	Sociocultural myths	Increased risk of cancer
		Infertility
		Delayed periods
		Early menopause
		Collection of dirty blood inside the uterus
		Side effects like hair loss, weight gain, alter women's sexual behavior, deformed babies

**Table 4:** Suggested solutions by the key informants to address the barriers to usage of IC (n=7) ASHA: Accredited Social Health Activist, ANM: Axillary Nurse and Midwife, RMNCH+A: Reproductive, Maternal, Newborn, Child Health and Adolescents, ANC: Antenatal case, IEC: Information, Education and Communication, IC: Injectable Contraceptives

Themes	Solutions
Individual-level barriers	Sensitization of eligible couples through informed choices of contraception by ASHA/ANM, RMNCH+A counselors. (counseling eligible couples, ANC clients, and postpartum women)
	Awareness and counseling on side effects through mass media, display of IEC tools in public places, and distribution of pamphlets and role play
	Creating support groups for reassurance and promoting self-help groups for health discussions on contraception
	Toll-based free helplines for doubts clarification
	Counselling in follow-up visits and addressing their concerns
Family-level barriers	Involving men and family members in contraceptive counseling
	Sensitization of women’s rights to choose contraception
Economic related barriers	Incentive-based follow-up approach (incentives can be given to motivator and user)
	Universal health insurance covering the treatment of family planning-related side effects
Sociocultural barriers	Community-level awareness through health education
	Positive deviance approach (promotive counseling through real IC users)
Community-level barriers	Community engagement and demand-generation activities
	Periodic training of service providers for quality services
	Training for the skilled service providers. (Motivating the clients, quality counseling regarding the side effects, and referral services for managing the side effects)
	Monitoring the side effects and follow-up of the clinical profile of contraceptive users including anthropometry
	Module-based teaching to grass field workers
	Sensitizing private sectors and public-private partnerships on the delivery and treatment of contraception

**Figure 1:**
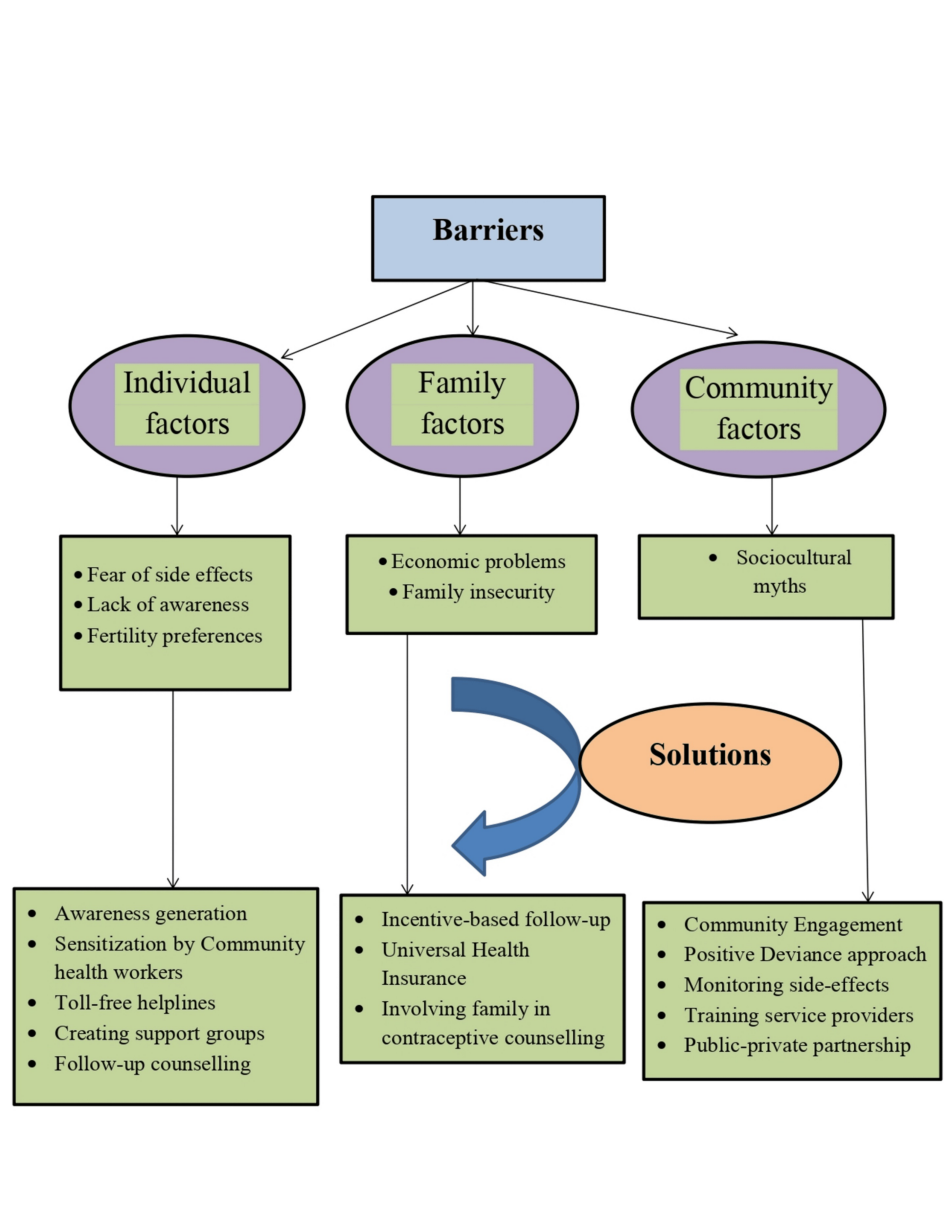
Thematic flow diagram of barriers and solutions to the utility of injectable contraception

The overall thematic framework of barriers and solutions has been depicted in Figure 1.

It has been described under three broad themes, namely, individual, family, and community-level barriers. The statements in italics were the direct verbatim of the participants.

Theme 1

Individual-Level Barriers

The side effects associated with IC and lack of awareness were the barriers perceived by the majority of the beneficiaries. In our study, we found that in addition to the menstrual side effects, mood swings and lethargy due to heavy menstrual bleeding also led to the rate of discontinuation.

Since it is a new method of contraception, women residing in rural areas were not aware of the choice of IC, which adds to the barriers.

Three users said, *“I have been asked to put IUCD after my first childbirth, and I didn’t know about the injections earlier”.*

Around six users said, “*Instead of taking this injection periodically, once every three months, I am willing to do family planning, which will be the permanent solution as it causes period problems.*”

Theme 2

Family-Level Barriers

Lack of support from family members and economic problems had a negative impact on the continuation of IC.

 One user said, *“I am not able to do my daily routine household activities due to heavy bleeding and difficulty taking care of my child because of lethargy, this is affecting our family's happiness”.*

Another user quoted that, *“My husband is a daily wager; engaged in work; he is scolding me for going to the hospital to treat the side effects periodically”.*

Most of the users were from below the poverty line; they found difficulty handling the medical expenses due to increased healthcare visits to overcome the side effects.

Around nine beneficiaries said, *“After taking this injection, I have increased flow during periods for the past 3-4 months. The doctor told me to take an iron injection; I am not affordable because my husband is a daily wage”.*

Theme 3

Community-Level Barriers

Socio-cultural factors include various myths and misconceptions about the usage of IC, which might lead to infertility, delayed periods, and early menopause.

One user said, *“My neighbor told me that taking this type of injection would affect childbirth”*.

Another user said, *“My in-law told me that this injection would lead to a deformed baby”.*

## Discussion

The present study found that lack of knowledge about IC, side effects associated with IC, lack of support from family, and socio-cultural misconceptions were the perceived barriers to IC usage. Key informants suggested community-level awareness generation, counseling, and managing side-effects in the follow-up period, family involvement in reproductive health, a positive deviance approach, periodical training for service providers, and a public-private partnership on the delivery of IC. An incentive-based follow-up approach and health insurance were proposed to address financial barriers.

In NFHS-5, about 62% of users were informed about the side effects of the current method of contraception. In our study, the acceptance and continuation rate was lower among women in the reproductive age group, mainly due to the side effects of injectable contraceptives, which was one of the unaddressed primary barriers identified for poor compliance. This aligns with most of the previous studies reported from eastern India [13], Haryana [14], and Karnataka [15]. Similarly, studies by Divya V et al. [16] and Mane N et al. [17] also found that side effects were the major reason for loss-to-follow-up. In our findings, around 37.5% of women discontinued after the initial dose, 18.75% in the second, and 43.75% had poor compliance after the third dose. Studies by Michelle Fonseca et al. [18] found that nearly 73% of patients had a loss of follow-up after the first dose, which was higher than our findings. Sirisha et al. [19] found that 58.5% discontinued after the initial two doses and 70.3% adopted tubectomy. However, in studies conducted by Nautiyal et al. [20] and Shilpa et al. [21], it was shown that injectable contraceptives were preferred by women, especially during their lactation period, due to their convenience and no effect on lactation. Henceforth, appropriate selection of eligible clients, counseling of side effects at the time of choosing contraception as well as during follow-up visits, and prompt management of side effects could increase the acceptance of contraceptives as suggested by healthcare workers.

In addition to the side effects, knowledge about the modern method of contraception was lacking in the community. It has been found that uneducated rural women of low socioeconomic status do not prefer modern methods of contraception [22]. However, clients value input from health workers in contraceptive counseling, leading to informed choices and improved user satisfaction [23]. Therefore, information about the benefits and effectiveness of ICs can be promoted through frontline health workers and mass media [24].

In our study, we encountered a lack of support from the partner and mother-in-law as a barrier to IC use at the family level. A Gambia study by Barrow et al. [25] stated that refusal by spouses was one of the ultimate causes of dissatisfaction with IC usage. A study conducted by Pradhan MR et al. [26] and Char A et al. [27] found that mothers-in-law influence women’s contraceptive behavior. Although women’s autonomy is crucial in reproductive choice, the involvement of men in joint decision-making has a significant influence on contraceptive communication among couples, which increases uptake and lowers discontinuation rates [28]. Key informants suggested women's empowerment and couple-based contraceptive counseling, encouraging family members to participate and support women's reproductive health care services during field visits.

Economic challenges such as out-of-pocket expenditure for medical appointments and the financial burden of managing side effects associated with DMPA were another obstacle found in our study. In India, contraceptive services are free of charge in public health facilities, whereas economic disparity exists in access to contraceptive services in the private sector [29]. Key informants indicated universal health insurance covering treatment costs and outpatient expenses associated with side effects of contraceptive usage. Encouraging public-private partnership contributions in the management of side effects can improve the acceptability of modern methods of contraception. They also recommended incentive-based follow-up strategies (in the form of money or ration) for users and motivators to encourage women who have completed DMPA doses during their sequential follow-up visits.

The strength of the study lies in its qualitative approach, which allowed us to explore the discontinuation of injectable contraception from multiple perspectives. The in-depth interviews provided a deeper understanding, capturing the lived experiences and the contextual nuances. By engaging with beneficiaries and stakeholders in their natural settings, we identified multifaceted barriers ranging from side effects to family dynamics, which helped us gain insights into the local community's perceptions and practices related to contraceptive use. Triangulating different data sources, investigator triangulation involving multiple researchers independently in content analysis, and the intercoder agreement strengthen the validity of the study.

While our study focused on injectable contraceptive users within a single center and specific reproductive age group, its generalizability to the broader community is limited. However, the findings offer valuable insights for targeted field interventions to enhance knowledge and promote injectable contraception. To improve generalizability, future multicentric studies are recommended with diverse populations based on geographic, educational, and socioeconomic backgrounds. Further combining quantitative and qualitative approaches and considering husband perspectives can provide a comprehensive understanding of the role of family in contraceptive usage. Reporting of side effects and follow-up of longitudinal data can assess trends and long-term effects on the population.

## Conclusions

Awareness from service providers influences the usage of injectable contraception. Acceptability could be improved by contraception information, quality counseling, regular follow-up, and effective management of contraceptive side effects. Devising methods of contraception that are free from side effects and universal health insurance are the needs of the hour to improve reproductive health.

## Appendices

Appendix 1

Interview Guide

Title: Acceptors, Barriers and Solutions to the Utility of Injectable Contraception

Briefing:

Hello Sir/Madam, I am glad to meet you here.

Myself a Doctor from the Department of Community Medicine at Sri Manakula Vinayagar Medical College and Hospital. I am doing my short study on injectable contraception. So, I want to ask you some questions related to injectable contraception - Depot Medroxy Progesterone Acetate (DMPA). I will add some probing questions in between when I ask these questions to get more information. Please feel free to tell me. This is not a test and there is no right or wrong answer. If this time is convenient for you to answer my questions, then can I proceed with it?  The whole interview will be audio-recorded if you give informed consent. Thanks!

Tell us something about yourself.

Name:                               Age:                                   Education:

Occupation:                      No. of months/years of Injectable contraception usage:

Opening question:

1. How did you come to know about injectable contraception?

Specific question:

1. What are the favoring factors that made you choose injectable contraception?

2. What according to you will be the side effects of using injectable contraceptives?

3. What according to you will be the possible barriers to using the injectable contraceptive?

4. Can you suggest possible solutions to overcome the above-listed barriers?

Exit question:

1. Is there anything else that you would like to add some more information?

Debriefing:

So according to you, the favoring factors, side effects, barriers, and suggested solutions are to be briefed to the participants. Thank you for sparing your time and sharing your valuable experiences. Thank you!
